# Monolithic 1 × 8 DWDM Silicon Optical Transmitter Using an Arrayed-Waveguide Grating and Electro-Absorption Modulators for Switch Fabrics in Intra-Data-Center Interconnects

**DOI:** 10.3390/mi11110991

**Published:** 2020-11-03

**Authors:** Uiseok Jeong, Dong Ho Lee, Kyungwoon Lee, Jung Ho Park

**Affiliations:** 1 School of Electrical Engineering, Korea University, Anam-Dong, Seongbuk Gu, Seoul 02841, Korea; jusjjang@korea.ac.kr; 2 MClavis Co., Ltd., Dongsan-ro, Seocho-Gu, Seoul 06784, Korea; dhlee7902@gmail.com; 3 Institution of Convergence Technology KT, Taebong-ro, Seocho-Gu, Seoul 06764, Korea; kyungwoon.lee@gmail.com

**Keywords:** silicon, optoelectronics, waveguide, electro-absorption, modulator, transmitter, AWG

## Abstract

In this study, we propose an eight-channel monolithic optical transmitter using silicon electro-absorption modulators (EAMs) based on free-carrier injection by Schottky junctions. The transmitter consists of a 1 × 8 silicon arrayed-waveguide grating (AWG) and eight 500-μm-long EAMs on a 5.41 × 2.84 mm^2^ footprint. It generates eight-channel dense wavelength-division multiplexing (DWDM) outputs with 1.33 nm channel spacing (Δλ) in the C-band from a single broadband light source and modulates each channel with over 3 dB modulation depth at 6 V peak-to-peak. The experimental results showed that the feasibility of a homogeneous silicon DWDM transmitter with a single light source for switch fabrics in intra-data-center interconnects over heterogeneous integration with regards to more complementary metal–oxide–semiconductor (CMOS) compatibility.

## 1. Introduction

Intra-data-center interconnects (up to 10 km) play a key role in determining the performance and scalability of these data centers. Intra-data-center traffic has been growing at a compound annual growth rate (CAGR) of 27% between 2015 and 2020 [1], mostly due to video services and associated machine-learning applications. Notably, cloud traffic has nearly quadrupled, representing 92% of data-center traffic in 2020 [2]. As the number of hosted applications and the amount of traffic in data centers continuously grow, the intra-data-center network faces the problems of power consumption and high cost created by the increasing traffic. Researchers are now looking for an interconnect solution that supports an ever-larger number of servers at an ever-higher bit rate [3]. Compared with electrical interconnects, optical interconnects offer the key merits of a high data rate, low latency, and a compact size. They have been viewed as a promising way to overcome the bottleneck of traditional electrical links in short-reach data-intensive applications regarding data centers.

Most of today’s commercial short-reach (<300 m) optical interconnects employ GaAs-based vertical-cavity surface-emitting lasers (VCSELs) emitting at 850 nm. VCSELs commonly apply a direct modulation scheme as a single-wavelength point-to-point photonic link. Recently, discrete multitone (DMT) modulation schemes have achieved 161 and 135 Gb/s transmission over 10 and 550 m multimode fiber (MMF), respectively [4]. This would impose a high price premium on VCSEL wavelength selection and complicate fiber coupling schemes for MMF. Moreover, it could significantly increase cost, footprint, and power consumption [5].

The introduction of wavelength-division multiplexing (WDM) functionality in intra-data-center interconnects plays a key role in overcoming VCSEL-based limitations and achieving cost-effective operation and low power consumption. To process a large amount of intra-data-center traffic in terms of switch architecture level, the large-scale optical circuit architecture networking solutions have been proposed, adopting wavelength routing that relies upon laser- or filter-based wavelength tunability [6]. In the case of optical packet switches (OPSs), bufferless OPSs using the wavelength conversion to solve the output packet contentions offer power consumption reduction [7]. Similarly, the use of integrated optical transport network (OTN)/WDM switch architecture was cost-effective, with low power consumption compared to conventional OTN switch architectures [8]. For this reason, WDM-based switch architecture is very important for the enhanced performance of intra-data-center interconnects with reference to cost and power consumption.

Currently, photonic integration circuits (PICs) have been developed in a much more compact, efficient, and cost-effective way by integrating the required WDM functionality on a single chip. The PICs can significantly reduce the device sizes and simplify the packaging procedures for intra-data-center applications. In regards to this, silicon photonics is seen as one of the most promising techniques due to its extra benefits of utilization of the existing complementary metal–oxide–semiconductor (CMOS) infrastructures and integration with microelectronics [9,10,11]. There are hybrid silicon PICs and monolithic silicon devices that enhance the characteristics considered to be key requirements for intra-data-center applications. Various hybrid PICs have been developed to demonstrate coherent transmitters and receivers. For example, silica or silicon planar photonic circuits have been copackaged with LiNbO_3_ [12] or InP modulators [13] for transmitters or assembled with Ge photodetectors [14,15,16] for receivers. Although hybrid PICs drive toward realizing low power consumption and direct modulation, the expensive packaging cost and device sizes in terms of CMOS compatibility still remain the major concerns [5]. Monolithic silicon PICs are commonly used in configurations of high-port-count N-by-N switch fabrics using Mach–Zehnder (MZ) interferometers and ring resonators as switch elements, leading to higher-order modulation formats for high data rate [3,17]. These modulators are still a concern as MZ type modulators require longer modulation length and larger driving voltage for modulation efficiency. While ring-resonator-type modulators reduce the modulation length, they increase the sensitivity to temperature, which could significantly increase cost, footprint, and power consumption with respect to modulator performance [18]. The electro-absorption modulator (EAM) is a good candidate as a switch element for optical modulation, providing a small device footprint, high speed, low driving voltages, and broad optical bandwidth operation. However, many EAMs based on silicon depend on SiGe multiple quantum wells (MQW) or III-V hybrid structure or graphene for variation of absorption coefficient (α;). They suffer from complex designs and fabrications [19,20]. In the context of laser integration into monolithic silicon PICs systems, a number of options are available for the choice of an adequate light source. One possibility is to use a single broadband light source in place of laser arrays for the feasibility of cost-effective and low-power-consumption switch fabrics in intra-data-center interconnects.

In this study, we propose a monolithic silicon optical transmitter consisting of an arrayed-waveguide grating and eight EAMs for switch fabrics in intra-data-center interconnects. Unlike the reported WDM devices with the arrayed-waveguide gratings [21], Mach–Zehnder interferometers [22], ring resonators [23], directional couplers [24], or an array of single-wavelength lasers [25], we incorporated homogeneous EAMs based on free-carrier plasma dispersion effect (FCPD) and a 1 × 8 arrayed-waveguide grating using star couplers. An arrayed-waveguide grating (AWG) of eight channels with 1550 nm center wavelength (λ_c_) and 1.6 nm channel spacing (Δλ) was designed. The device also employed a compact electro-absorption modulator utilizing a Schottky diode. Optical modulation using a Schottky diode was achieved by the intensity change of guiding light due to the free-carrier absorption in the semiconductor to change its absorption coefficient, but not conventional interference effects. Each channel output of the fabricated device on a 5.41 × 2.84 mm^2^ footprint showed −47 dBm with over 3 dB modulation depth at 6 V peak-to-peak, experimentally. It showed the feasibility of achieving cost-effectiveness and small footprint using homogeneous silicon EAMs and an AWG with a single broadband light source with regards to more CMOS compatibility. Section 2 describes the device design and fabrication. The characteristics of the fabricated silicon transmitter chip are presented in Section 3.

## 2. Device Design and Fabrication

### 2.1. Silicon Waveguide and Electro-Absorption Modulator Using a Schottky Diode

**Figure 1:**
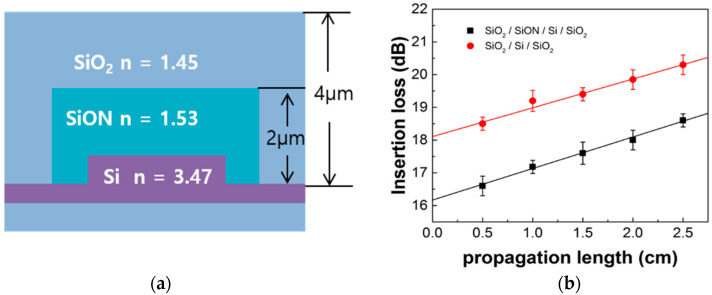
(**a**) The optical waveguide cross-section; (**b**) the insertion losses of two waveguides (square: SiO_2_/SiON/Si/SiO_2_ waveguide; circle: SiO_2_/Si/SiO_2_ waveguide).

First, the silicon rib waveguides were designed on the 250 nm silicon-on-insulator (SOI) platform as shown in Figure 1a. The rib waveguide dimensions were a height of 250 nm, an etch depth of 150 nm, and a width of 4.8 μm for low-loss waveguiding in the C-band. We added a SiON layer to passivate the device and reduce coupling loss. For comparison between waveguides with and without the SiON insertion layer, the optical power from each waveguide was measured at the length of the waveguides from 5 to 25 mm by a cut-back method as shown in Figure 1b. Propagation loss was 0.92 and 0.9 dB/cm for the waveguides with and without the SiON insertion layer, respectively. However, the coupling loss was 16.2 dB/cm for the waveguide with the SiON insertion layer and 18.1 dB/cm that without. The waveguide with the SiON insertion layer had a coupling loss of 1.9 dB, which was lower than that without the SiON insertion layer. The incident light was mostly confined to the SiON insertion layer having a higher refractive index than the refractive index of the SiO_2_ cladding. As a result, a larger percentage of the main optical field is concentrated in the core, reducing the coupling loss [26,27,28].

**Figure 2:**
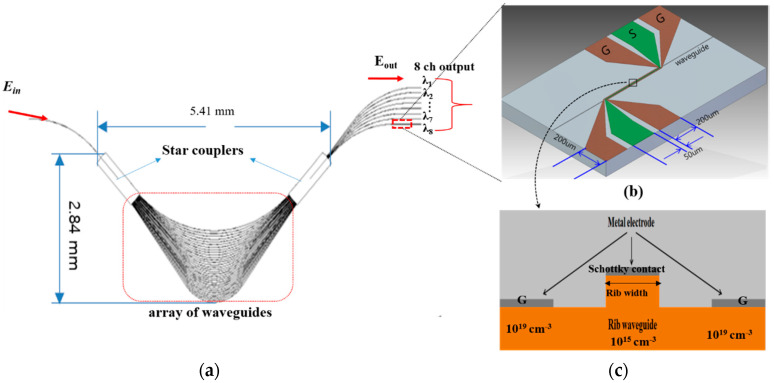
(**a**) Schematic view of 1 × 8 channel silicon transmitter with electro-absorption modulators (EAMs); (**b**) top view of the silicon EAM with coplanar waveguide (CPW); (**c**) cross-section view of the EAM.

Based on the measured silicon waveguide characteristics, we designed the silicon EAM without utilizing conventional interference effects, which are used in Mach–Zehnder and ring resonator optical structures to reduce the device size. The proposed EAM had lateral metal–semiconductor (MS) junctions to maximize the free-carrier injection and depletion by a Schottky contact on the rib waveguide center. Optical modulation was achieved by the change in intensity of guiding light due to free-carrier absorption in the semiconductor to change its absorption coefficient. The change of absorption coefficient due to the changes in the electron and hole concentration was calculated using the following Equation (1) [29]:

Δα_e_ = 8.5 × 10^−18^ × ΔN_e_, Δα_h_ = 6 × 10^−18^ × ΔNh (1)

where Δα_e_ and Δα_h_ are the changes in absorption coefficients resulting from changes in the free-electron and free-hole carrier concentrations (ΔN), respectively. Based on Equation (1), the n+–n–n+ junction was more efficient for modulation than the p+–p–p+ junction. The silicon EAM was designed on a rib waveguide of 4.8 μm width embedded with a lateral n+–n–n+ junction as shown in Figure 2c. The sides were heavily doped with 10^19^ cm^−3^ phosphorus to enhance the optical absorption change in the center. The center of the rib waveguide was lightly doped with 10^15^ cm^−3^ phosphorus. This design allowed a large overlap between the optical mode and carrier density variations in the center of the waveguide. The optical modulation depth was estimated by the following Equation (2):

Modulation depth [dB] = 10 log (T_0_/(T_0_ · exp(Δα_e_ · L)) (2)

where L is the modulation length and T_0_ is the initial propagated optical intensity. According to Equation (2), the intensity modulation of the propagating light depends on the modulation length (L) and optical absorption coefficient change (Δα_e_) due to the change in the free electron concentration. For a 500 μm modulation length with 3 V_pp_ of driving voltage, the simulation results demonstrated a 3.3 dB modulation depth, which meets the requirement for short-reach optical interconnects in the C-band [30]. In addition, the EAM employed a traveling-wave-type electrode running on the modulation length—known as coplanar waveguide (CPW) electrode—as shown in Figure 2b.

### 2.2. Silicon DWDM Transmitter for Switch Fabrics in Intra-Data-Center Interconnects

For use with a single incident light source operating in the C-band, we introduced a silicon 1 × 8 AWG using star couplers as shown in Figure 2a. An optical field (E_in_) containing multiple wavelengths (λ_1_ ~ λ_n_) was coupled into the input waveguide. In the input star coupler, the light beam became divergent and was coupled into the array of waveguides. Their length difference (ΔL) was such that their optical path length difference equaled an integral multiple of the center wavelength, ΔL = m·λc/n_eff_. The fields in the individual arrayed-waveguides arrived at the input aperture of the output coupler with equal phases. In the output star coupler, the light beam interfered constructively and was focused at one single focal point of the focal line. The outgoing beam was shifted along the image plane. Each output waveguide was placed along this image plane for capturing a different wavelength in the spatially separated spectrum. The AWG footprint reduction allowed the integration of more components on a single chip. However, the fabrication tolerances could be a concern. To optimize footprint and fabrication tolerances, the design parameters were chosen as listed in Table 1 so as to fabricate the device with a smaller footprint. Particularly, we focused on AWG for dense wavelength-division multiplexing (DWDM) with 1.6 nm narrow channel spacing widely used in short-reach optical interconnects. For the AWG with narrow channel spacing (Δλ < 1.6 nm), the spectral full width at half maximum (FWHM) is the important parameter compared to the crosstalk [31]. The waveguide separations (D_i_ and D_o_) were set as variables from 4 to 10 μm for narrow FWHM and small crosstalk.

**Figure 3:**
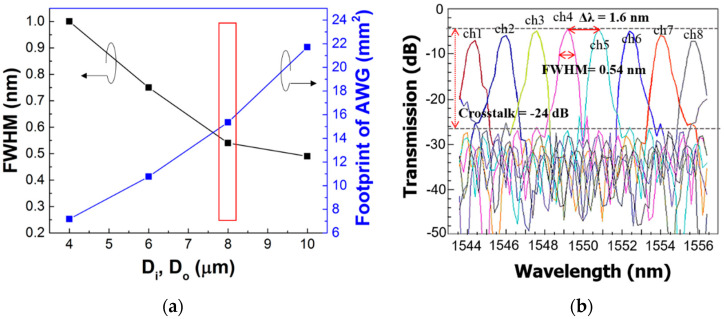
Simulated optical spectrums of AWG: (**a**) trade-off between FWHM and footprint of designed AWGs; (**b**) transmission spectrum for optimized waveguide separation (Di, Do) of 8 μm.

For the evaluation of the designed AWG, the characteristics of wavelength distribution were simulated with BeamPROP software, which is based upon beam propagation method (BPM) analysis. The crosstalks were approximately −24 dB for the designed AWGs. The FWHMs were 1, 0.75, 0.54, and 0.49 nm for the 4, 6, 8, and 10 μm waveguide separations, respectively. Although the FWHM reduced as the waveguide separation increased, the footprint of AWG increased almost linearly with the waveguide separation, as shown in Figure 3a.

For a suitable footprint and narrow FWHM of AWG, D_i_ and D_o_ were chosen as 8 μm. The footprint of the designed AWG was 5.41 × 2.84 mm^2^. As shown in Figure 3b, the AWG operates in the range of 1542 to 1556 nm with 1.6 nm channel spacing (Δλ), and it can be applied to a silicon transmitter for short-reach optical interconnects in the C-band. Finally, the Si transmitter was designed by inserting the proposed EAM into each output channel of the AWG.

**Figure 4:**
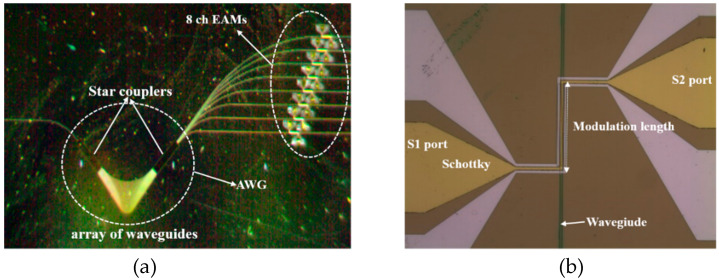
Microscope images of fabricated devices: (**a**) 1 × 8 channel silicon transmitter with EAMs; (**b**) a silicon EAM with a modulation length of 500 μm.

The device was fabricated using a 250 nm silicon-on-insulator (SOI) wafer, which had 2-μm-thick buried oxide. During the monolithic fabrication using the CMOS process, the reduction of sidewall roughness was intensively studied for low-loss silicon waveguides. As a result, the trim etching technique with SiO_2_ hard mask was used in the RIE process [32]. For the ion implantation process, the SiO_2_ layer with a thickness of 600 nm was deposited on the SOI wafer. The SiO_2_ hard mask was patterned on the top silicon layer through a photolithographic process, and phosphorus ions were implanted to both sides of the ribs to achieve a doping concentration of 10^19^ cm^−3^. Then, the 250 nm top silicon layer was etched by RIE using SF_6_ and CF_4_ gases to form the silicon waveguide and AWG. Both sides of the rib were etched to 150 nm at a rate of 1 μm/min using RIE with the SiO_2_ mask. The SiON layer was deposited to passivate the device and reduce coupling loss as mentioned in Section 2.1. SiO_2_ was deposited on the entire surface of the device as a top cladding. Holes were formed on the top silicon layer for the metal contacts, and aluminum (Al) electrodes with thicknesses of 200 nm were formed. Annealing was performed at 700 °C to form an ohmic contact. Finally, a gold (Au) electrode with a thickness of 200 nm was formed for the n-type Schottky contact. The fabricated eight-channel silicon optical transmitter was as shown in Figure 4a. All the main parts of the fabricated optical transmitter were made of silicon monolithically, as compared to the many reported works [33].

## 3. Experimental Results

### 3.1. Characteristics of a Silicon EAM Using a Schottky Diode

**Figure 5:**
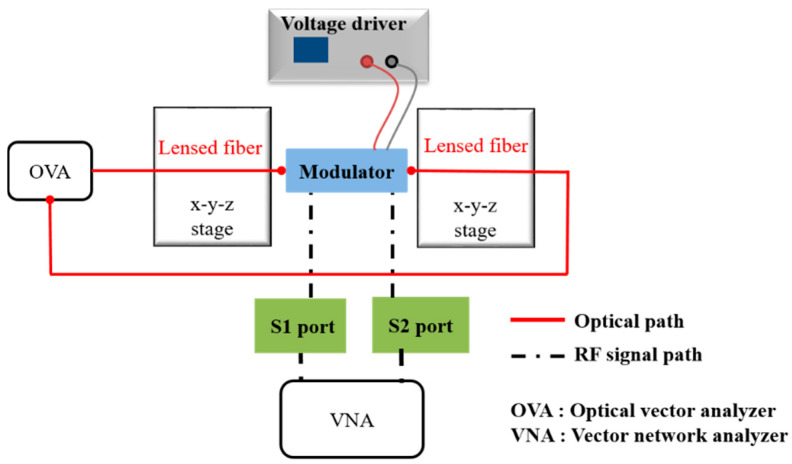
Schematic view of the experimental setup employed to characterize the Si EAM.

To examine the intensity modulation of the silicon EAM with a modulation length of 500 μm as a function of wavelength, we used an experimental setup as shown in Figure 5.

First, continuous-wave (CW) light from an optical vector analyzer (OVA) was coupled into the device using a 5-μm-diameter lensed fiber, while the OVA (Luna Technology OVA5000) was controlled by a polarization controller (PC).

**Figure 6:**
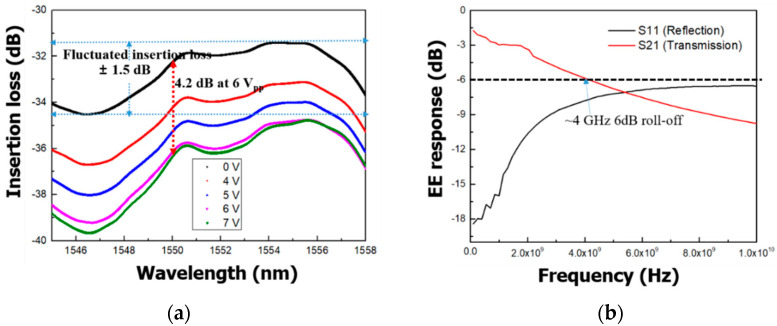
Experimental characteristics of a silicon EAM: (**a**) insertion losses and modulation depths of the EAM; (**b**) electrical response.

We measured insertion losses and modulation depths based on the bias using an OVA wavelength ranging from 1542 to 1558 nm as shown in Figure 6a. The insertion loss of an EAM with a 500 μm modulation length was −33 ± 1.5 dB at 0 V. The fluctuation of insertion loss as a function of wavelength was ±1.5 dB, which is sufficient for short-reach optical interconnects [34]. In addition, the modulation depth was measured as 4.2 dB for a 500 μm modulation length with 6 V_pp_ driving voltage in the wavelength range of 1542 to 1558 nm. The value of the modulation depth of the EAM was consistent in the operating wavelength range. Since the free carriers associated with the absorption coefficient were fully injected into the center of the waveguide at 6 V, the modulation depth reached its saturation. As mentioned in Section 2.1, the measured modulation depth of over 3 dB is sufficient for use as optical interconnects in the C-band. The experimental RF electrode output S21 (dB) showed a 6 dB electrical bandwidth of ~4 GHz as shown in Figure 6b. The traveling-wave-type electrodes enabled the modulator to operate to up to 4 GHz. The RF 6.4 dB bandwidth is close to the EO response 3 dB bandwidth without velocity mismatch and other nonideal RF effects (such as reflection or multimodal behavior) [35].

### 3.2. Characteristics of a Silicon Transmitter with Eight EAMs

**Figure 7:**
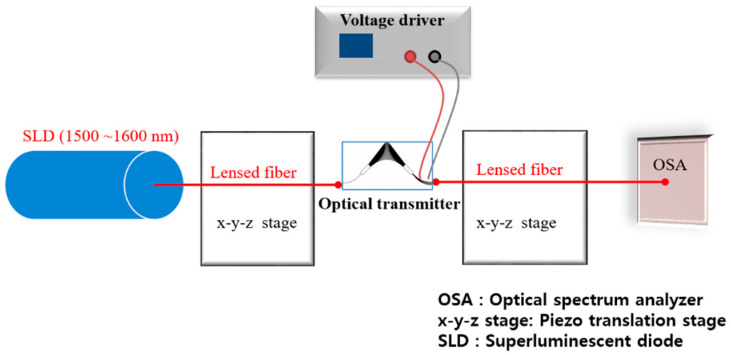
Schematic view of the experimental setup employed to characterize the Si transmitter.

**Figure 8:**
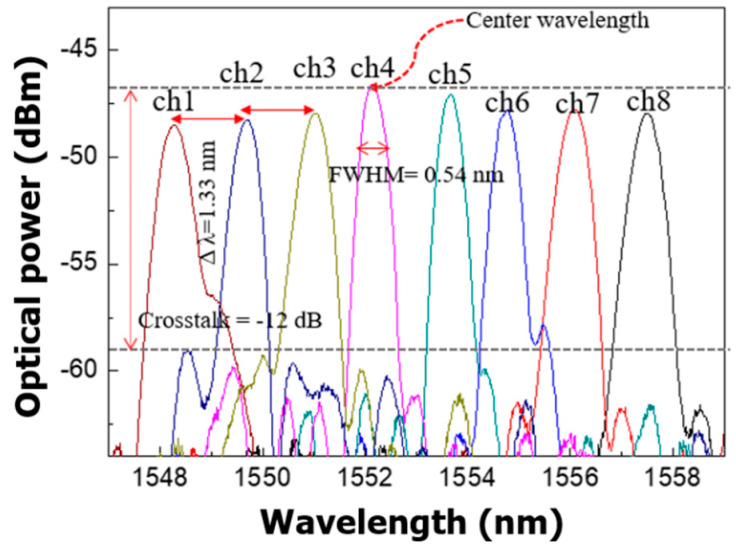
The experimental optical spectra of the silicon transmitter.

To evaluate the optical spectrums of the 1 × 8 silicon transmitter using the EAMs with a 500 μm modulation length, a superluminescent diode (SLD, Thorlabs S5FC1005S-A2) light source (1500–1600 nm wavelengths) and an optical spectrum analyzer (Ando AQ6319) were used as shown in Figure 7. First, we measured the optical spectrums as shown in Figure 8 below.

The measured FWHM was 0.54 nm, which is in good agreement with the simulated result. The maximum optical power from each channel was approximately −47 dBm using a 2.5 mW SLD as a light source. The total insertion loss was approximately −51 dB, including the coupling loss and the transmitter loss. The center wavelength (λ_c_) was 1552.8 nm, and channel spacing (Δλ) in the wavelength was 1.33 nm. The differences in the center wavelength and the channel spacing were 2.8 and 0.27 nm, respectively, compared to the simulated results in Figure 2b. The crosstalk of the transmitter was measured as −12 dB; however, it depends on the quality of the fabrication process, device size, channel spacing, number of channels, etc. Although the crosstalk increase is mainly due to the phase errors caused by waveguide fabrication imperfections [36,37], measured crosstalk of −12 dB is acceptable for optical interconnects [31].

**Figure 9:**
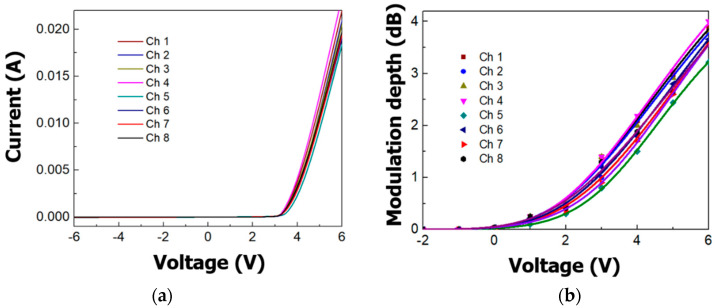
The experimental correlation of current–voltage (I–V) characteristics and modulation depths: (**a**) I–V characteristics; (**b**) modulation depths of the silicon transmitter.

The current–voltage (I–V) characteristic of the Schottky junction at each EAM was measured for the bias voltage from −6 to 6 V as shown in Figure 9a. In the reverse bias, a very small leakage current of 10^−7^ A or less was observed. According to Equations (1) and (2), a small leakage current has no significant effect on the change of the absorption coefficient in the rib center. In the forward bias, an average current of 20 mA flows at 6 V. A large current can enhance the modulation depth as a result of the optical modulation mechanism of the free-carrier plasma dispersion effect in silicon [20,29]. The results are consistent with the Schottky I–V characteristics due to the injection and depletion of free carriers through the change of bias. The measured modulation depths of eight channels were from 3.22 to 4 dB as shown in Figure 9b. These results showed modulation depth characteristics of the single EAM similar to those mentioned in Section 3.1. Despite the small variation in modulation depth of each channel due to the fabrication tolerances, the 1 × 8 channel transmitter based on silicon EAM shows the possibility of operation for switch fabrics in intra-data-center interconnects in terms of cost-effectiveness and small footprint. Our device similarly demonstrated 6 V_pp_ driving voltage and small footprint as compared with an N-by-N DWDM transmitter with MZ modulators as switch elements with regards to more CMOS compatibility [38].

## 4. Conclusions

In this study, we proposed an eight-channel silicon DWDM transmitter on a 5.41 × 2.84 mm^2^ footprint with a single SLD as a light source. The device homogeneously integrated a 1 × 8 silicon AWG using a 32-waveguide array, two star-couplers, and eight silicon EAMs based on free-carrier injection and depletion by Schottky diodes. We successfully achieved the optical intensity modulation of the device through free-carrier absorption change, rather than through conventional interference effects. Therefore, it did not require a Mach–Zehnder-type structure, which uses a large footprint to obtain modulated signals. The AWG separates eight DWDM wavelengths from an output of SLD with 0.54 nm of FWHM and 1.33 nm of channel spacing at 1552.8 nm of center wavelength. Each 500-μm-long EAM demonstrated over 3 dB modulation depth at 6 V_pp_ from 1542 to 1558 nm. This study proposed and demonstrated the use of a DWDM transmitter for switch fabrics in the intra-data-center interconnects using a homogeneous integration of Si AWG and Si EAMs. The proposed device can be a promising alternative for DWDM in short-reach optical interconnects over heterogeneous/hybrid integration with regards to more CMOS compatibility with a single light source. It is expected to contribute to switch fabrics in intra-data-center interconnects in terms of cost-effectiveness and small footprint.

## Figures and Tables

**Table 1:** The designed parameters and values for silicon arrayed-waveguide grating (AWG).

Parameter	Description	Value
w	width of rib waveguide	4.8 μm
λ_c_	center wavelength	1.55 μm
Δλ	channel spacing (in wavelength)	1.6 nm
M	number of waveguides in array	32
N_ch_	number of output channels	8
R_aw1_	radius of i^th^ innermost waveguide bend in array	2500 μm
D_i_	waveguide separation at input circle	4, 6, 8, 10 μm
D_o_	waveguide separation at output circle	4, 6, 8, 10 μm
